# The role of local knowledge in enhancing the resilience of dinki watershed social-ecological system, central highlands of Ethiopia

**DOI:** 10.1371/journal.pone.0238460

**Published:** 2020-09-04

**Authors:** Mengistu Asmamaw, Seid Tiku Mereta, Argaw Ambelu

**Affiliations:** 1 Department of Environmental Health Science and Technology, Jimma University, Jimma, Ethiopia; 2 Department of Biology, Debre Berhan University, Debre Berhan, Ethiopia; Feroze Gandhi Degree College, INDIA

## Abstract

This study was designed to explore the role of local knowledge (LK) in enhancing the resilience of Dinki watershed in the central highlands of Ethiopia. The data were collected through focus group discussions, key informant interviews, and household surveys. The qualitative data were analyzed through thematic analysis. Whereas descriptive statistics and logistic regression were used for quantitative data analyses. The result showed that the majority of the respondents have local knowledge and used in their daily activities. Furthermore, the LK is reported to have the adaptive capacity in managing land resource degradation, treating disease, mitigating food insecurity, and enhancing social capital, among others. Likewise, the logistic regression analysis showed that age, gender, and education status of the household head were significantly correlated (p<0.05) to knowledge level, indicating their predictive power for the acquisition of LK. Accordingly, old-aged (60+ years) male respondents who attended primary education were higher to acquire LK than their counterparts. The result realized that the adaptive roles (land resource management, medication, climate change adaptation, etc.) of local knowledge systems could contribute to enhancing resilience. More importantly, the presence of social mechanisms is insurance to maintain LK. Thus, both intergenerational and intragenerational information gaps are filled with education and promotion on the roles of local knowledge systems. Besides, local-decision options should participate in custodians to share their experiences, that could contribute to sustaining ecosystem resilience.

## 1. Introduction

The local people have survived in harmony with their environment for millennia and acquired a cumulative body of knowledge that enabled them to adapt to and mitigate environmental disturbances [1]. In developing countries where the communities lack advanced technology to manage environmental disturbances [2], local knowledge systems are the major options to adapt to and mitigate with environmental disturbances [3]. However, the local knowledge systems have been often segregated from decision-making, which might be attributed to the failure history of most natural resource management programs [4].

The term local knowledge (LK) is defined in by various scholars. However, the concept by Berkes, Colding, and Folke [5] is usually cited as a common working definition, whichh is stated as

*“cumulative body of knowledge*, *practice*, *and belief*, *evolving by adaptive processes and handed down through generations by cultural transmission*, *about the relationship of living beings (including humans) with one another and with their environment”*.

Despite dichotomy across various scholars, terms like indigenous knowledge, traditional ecological knowledge, local knowledge, and eco-literacy are used interchangeably in this study and, which are options aimed to capture human-nature interaction [6].

The potential of local knowledge (LK) or local technical knowledge (LTK) in enhancing the resilience of socio-ecological can be viewed based on (i) their ability to enhance biological diversity, and (ii) their ability to manage environmental disturbances (example: soil erosion, disease, soil fertility, conflict management, food security, etc.) [5]. In terms of biodiversity conservation, although it is not their direct objective, local knowledge systems are effective in promoting species diversity, habitat heterogeneity, and has been used for nature conservation in various localities globally [1].

In a community-based conservation strategy (adaptive management), the resident communities have a diversity of cultures to sustain pristine habitats and contribute to conserving biological diversity and thereby resilience of the social-ecological system [7]. In this aspect, the cultural diversity improves the biological diversity, and the biological diversity enhances the resilience of the social-ecological system. Specifically, the knowledge-belief-practice model best explains the way human knowledge, and belief influences the natural environment [6]. Accordingly, when humans face with a disaster, they seek engagement with nature to demonstrate resilience [8].

The local knowledge systems are cost-effective and sustainable alternatives for rural development, and biodiversity conservation through their potential for natural resource management, health care, and disaster responses [1,3]. The potential of the LK to manage natural resources, treat human and animal disease, sustain social capital, among others indicates their adaptive potential to adapt to, and mitigate with the effects of environmental disturbances [9,3]. Effective natural resource and disturbance management practices will result in a sustainable ecosystem that resists and or recovers from environmental disturbances. Such a system is then resilient to environmental disturbances [5,10,11].

In the recent period of accelerated environmental degradation, inventory of indigenous practices is plausible to document valuable database on human-nature interactions. In this aspect, resilience-framework is sound to simultaneously analyze the human, and ecological dimensions of a system to draw integrated management options [12]. Moreover, the full resilience assessment involves searching for resilience assets and adaptive practices that could contribute to enhancing system resilience to adapt, learn, and renew the following disturbances [13]. In a local setting, resilience relies on the potential of local knowledge and institutions to reorganize and adapt to changes [12].

Resilience theory perceives local knowledge as experiential knowledge acquired over long-term experimentation, and as a means to respond to environmental disturbances [14]. Adaptive capacity is an action taken by the community in response to environmental disturbances, and the basis to acclimatize to environmental disturbances. However, impairment in adaptive capacity makes a social-ecological system vulnerable to surprises leading to erosion of the socio-ecological system [15].

Promoting local knowledge is recognized to be central to maintain human-nature interaction which is argued to be the critical source of resilience during a crisis [8]. However, empirical evidence on the performances of local knowledge in enhancing resilience to environmental disturbances are insufficient in Ethiopia and lacking in the study area. Besides, integrating knowledge from local and scientific sources is credible to formulate tangible outputs, interpret results, and basis to enhance social-ecological system resilience [7,8,16].

As a result, this study was designed to explore the resilience enhancing roles of LK, and determinant factors for the acquisition, and transmission of traditional knowledge, and practices in the Dinki watershed social-ecological system, central highlands of Ethiopia. Thus, the research questions were formulated as: (i) Are there local knowledge systems that are applied to manage local-level disturbances in this locality? (ii) How is the management and current utilization trends of local knowledge systems in this locality? (iii) Do local knowledge systems have adaptive roles in managing environmental disturbances? (iv) What are the social mechanisms that sustain local knowledge systems? (v) What factors determine the acquisition of local knowledge systems?

## 2. Materials and methods

### 2.1 The study area

**Fig 1:**
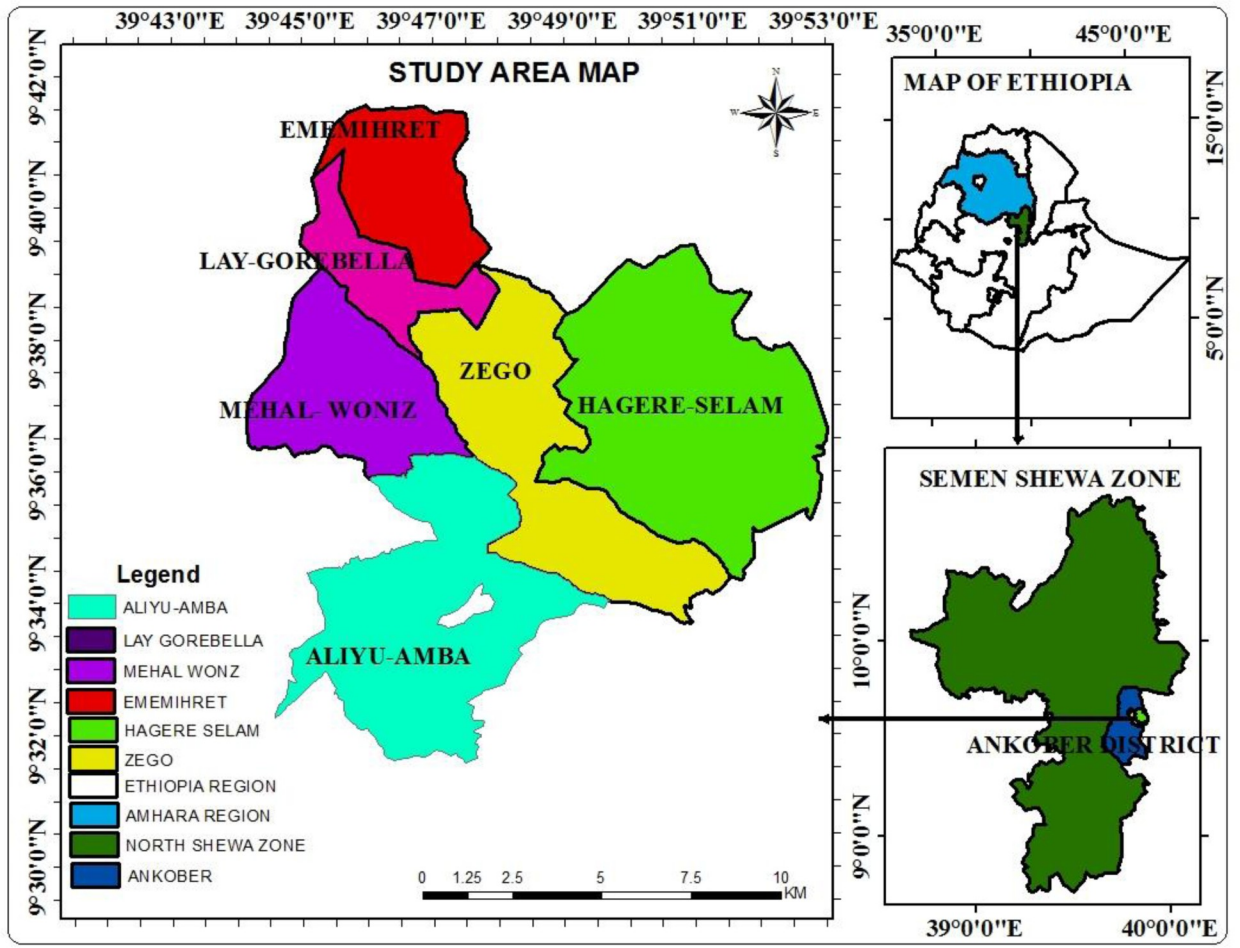
Location of Dinki watershed in the central highlands of Ethiopia [17].

The study area, Dinki watershed is found in the Ankober district, central highlands of Ethiopia. Gorobela, the city of the district, is located at 172 km north of Addis Ababa, the capital city of Ethiopia and 42 km east of Debre Berhan (the capital of North Shewa Zone). Hills and mountains constitute the majority of land area in the district (75%), whereas the rugged terrains and plain topography account for 17% and 8%, respectively. Dinki watershed is located between 9° 32' 30” to 9° 42ʹ 30" N latitude and 039° 41' 0" to 039° 53ʹ 0" E longitude. The altitudinal range of the watershed varies from 1,300 m above sea level (a.s.l.) near Addis Alem (at the downstream) to the 3,700 m a.s.l. at the Kundi Mountain (upstream). Dinki watershed is drained by a third-order Dinki stream and covers a total area of 16, 537 ha land area (Fig 1).

The Rainfall pattern is bimodal where some short and long-term rainy periods are recorded in March and in late June to September, respectively. A 30 year (1987–2016) of meteorological data showed a mean annual rainfall of 1,179 mm; where the mean minimum and maximum monthly temperatures were 6.47 and 19.99 ^º^C, respectively. The watershed is drained by a third ordered stream and has a total area of 16,537 ha [18].

### 2.2 Study design, source population, and sampling

A cross-sectional study design was used to collect samples from the highland, midland, and lowland agro-ecological zone of the Dinki watershed. The sources of the primary data include key informant interviews, focus group discussions, and a household survey. Whereas the secondary data, such as the climatic and agro-ecology related information was obtained from the national Metreological stations, Addis Ababa, Ethiopia, and Ankober district agricultural office, respectively. The study populations were the agricultural experts in the district and household heads or wives or any household member above 18 years living in Dinki watershed at least for a year.

Simple random and purposive sampling techniques were used to select households for household survey and interviews, respectively, whereas the agro-ecological zones were classified based on the Ethiopian agro-ecological zonation and information from the agricultural office of the Ankober district. Accordingly, the villages (the smallest admirative unit) and *gots* (smaller administrative unit than the village) included in the highland agro-ecology were the Ememihret, Lay-Gorobela, and upper parts of the Mehal-Woniz (Dens). The lower parts of the Mehal-Wonz (Lik-Marefiya), upper parts of Zego (Gedamoch), and the upper parts of the Hagere-Selam were included in the midland agro-ecology. Likewise, the lower parts of Zego and Hager-Selam were included in the lowland agro-ecological zones.

### 2.3 Data collection

A participatory Rural Appraisal approach using key informant interviews, focus group discussion, and household data were used to collect the data. Data on the local knowledge and practices were collected based on the knowledge-belief-practice analytical model, where a set of variables were used to collect the eco-cognitive, practical, and sociocultural practices [6]. Tools applied to collect local natural resource and disturbance management practices as well as sociocultural mechanisms were adopted and customized from literature [5,11,10]. The individual interview and focus group discussion data were collected during November 2017 and the household questionnaire data was carried out during February 2018.

#### Focus group discussion (FGD)

A total of four FGDs (two FGDs in the highland, and lowland, and two-gender segregated FGDs in the midland agro-ecological zones), each having 8–12 participants were carried out to explore the current management and utilization trends of local knowledge systems. Participants were selected from community leaders, elders, extension workers, resource conservation groups, youth, and women representatives. Issues on the basic conceptual knowledge (knowledge on system dynamics, climate condition, soil types, etc.), practical and sociocultural dimensions of local knowledge systems were considered. Such issues were raised to capture their knowledge on their environment as well as to document participants’ knowledge and participation status in practical (activities in agriculture, natural resource, and disturbance management practices) and sociocultural (practices in setting rules, rituals, cultural and spiritual activities, etc.) domains of indigenous knowledge systems.

#### Key informant interview

The same interview questions were used to conduct 15 face-to-face interviews involving various community members, such as religious leaders, watershed management group members, elders, youth, women as well as representatives from school and development agents. The interview aimed at documenting on the local-level environmental disturbance management practices. A snowball sampling technique was used to purposively select individuals who are assumed to have natural resource and disturbance management experiences. The focus group discussion and key informant interviews were guided by checklists, where the leading questions were raised by the interviewer (corresponding author) and allow the discussants and the interviewee to narrate as much as they can. Information redundancy was used as insurance for information saturation and the interviewer proceeds to the next question, and so on. During the discussion and narration, one filed assistant was assigned for open note-taking. Hence, the target populations were Amharic language speakers, all the communication was carried out in Amharic. The results were translated into English, organized, and summarized through content analysis. A total time range of 25 to 30 minutes was used to complete the individual interview. However, a single focus group discussion was lasted in a range of 60 to 90 minutes and extended up to 2 hours on occasions when participants are willing to debate [19,20].

#### Household data collection

Following the focus group discussions and key informant interviews as well as based on related literature, a household questionnaire was formulated. The questions framed based on ‘resilience-with-what?’ analysis (searching for local-level coping strategies to manage climate change extremes, food insecurity, soil erosion, etc.) was employed to document households’ local natural resource and disturbance management practices. The selection of respondents involves receiving N lists of households from respective village administrators, calculating the sample size n, providing of numeric number for every N households. Finally, the households from the list of the population were selected randomly using a lottery method [21]. The sample size determination followed the formula of small sample size population correction to improve the power of the statistical test following the prescription by Kothari [22] and Daniel and Cross [23] were used to determine sample size as follows:

$$n=\frac{N*p*q*Z^{2}}{e^{2}(N-1)+Z^{2}*p*q}$$

Where: n = sample size

Z = 95% confidence interval under the normal curve that is 1.96

p = 0.5 (proportion of the population to be included in the sample that is 50%)

q = None occurrence of event = 1–0.5; that is 0.5

N = size of population

e = Margin of error or degree of accuracy (acceptable error term) (0.05)

Therefore, a sample size of 294 respondents was obtained from a total of 1, 245 households. However, only 288 households (six respondents who did not fill the questions appropriately were excluded), of which 82 females and 206 males properly responded and returned the questionnaire, resulting in a 97.96% response rate.

The household data were collected by enumerators selected from agricultural extension workers in respective agro-ecology. A total of six enumerators (two in each agro-ecology) were involved in the household survey data collection. Before the actual survey, enumerators and supervisors were trained for three days on the general data collection techniques and context of the questions. Moreover, pilot tests were made on 5% of the total sample, which was not included in the final survey around the Gorobela village communities to familiarize the enumerators with the paper survey. Prior to the actual data collection, a consent letter which also approved this particular study was received from the Institutional Review Board (IRB), Institute of Health, Jimma University and submitted to the district administration office. A copy of this letter was distributed to each village administrator.

### 2.4 Data analysis

The qualitative data were summarized through thematic analysis, based on the knowledge-belief-practical analytical model, where eco-cognitive, practical, and sociocultural domains were the major themes and sub-sectioned into respective components and elements (Table 4). The quantitative data were categorized, coded, and simple descriptive statistics were used to compute percentages and frequencies. In addition to the knowledge level, the participation status of households, especially in practical and normative domains are assumed to indicate the acquisition of local knowledge systems. Accordingly, the average of the knowledge and participation status of indigenous knowledge was presented in tables and figures as well as variations in mean values of eco-cognitive, practical, and normative domains between the explanatory variables were computed. In effect, chi-square statistics were computed to explore the effects of a set of predictors on the knowledge level and participation status of practical and socioeconomic domains. The effect of age on the acquisition of the eco-cognitive domain was analyzed through one-way Analysis of Variance (ANOVA). Whereas, the gender-based variation in acquisition of the eco-cognitive domain was computed using the t-test.

A logistic regression model was used to compute the effects of sets of explanatory variables with the dichotomous dependent variable. This model is more flexible and appropriate for explanatory variables with discrete, continuous, and dichotomous nature. It is suited for the dichotomous outcome variable and preferred to discriminate analysis as it has no assumptions on predictor variables. All the statistical analyses were carried out using the IBM Statistical Product, and Service Solutions package [24].

### 2.5 Working hypothesis and variable specification

In this study, it has been hypothesized that farmers’ knowledge or awareness level of some disturbances as well as natural resources conservation practices are influenced by the joint effects of sociodemographic (age, gender, and education), resource endowment (farm size and farming experience) and environmental factors (agroecology). Prior to running the logit model, the variables were checked for multi-collinearity using Tolerance statistics and variance inflation factor (VIF). As well as Mahalanobis distance was computed to check outliers using the chi-square criteria of *X* ^2^ (6) = 22.46 at p = .001.

The logit model was used to analyze the independent variables that were hypothesized to be determinants for the awareness of indigenous knowledge. The dichotomous dependent variable, knowledge level, KnowLevel, was presented based on the knowledge-practice-belief analytical model [6]. Accordingly, the knowledge level was contextualized as eco-cognitive, practical, and normative domains. The basic conceptual knowledge of respondents to their environment and climate (such as local climate, biological organisms, soil type, land-use type, and ecosystem dynamics) were used in this study to capture their eco-cognitive domains.

In the second level of analysis, the respondents’ knowledge and participation status in agricultural and natural resource management practices as well as adaptive practices like medication, storage, etc., were used to explain the practical domains [25]. The practical dimension is where farmers use in their day-to-day livelihood activities, and their knowledge and participation statuses were explored [6]. Whereas respondents’ knowledge and participation status in sociocultural practices, such as the family knowledge, in making rules, rituals, cultural values, etc. were used to describe the sociocultural domains of the indigenous knowledge system (Table 4).The socio-cultural aspects of the IKPs are also termed as the normative domains [26].

The respondents were asked whether or not they are aware of, and participate in each of the parameters based on dummy (1 = yes;0 = no) questions. The positive answers from the 25 items (S1 Appendix) were converted into a 5-point scale and averaged based on cutoff 3 to get the dichotomous dependent variable, KnowLevel. The KnowLevel = 1 was designated for households who scored ≥3, 0 otherwise [27]. The candidate explanatory variables were extracted from intuition, theoretical explanations, and the authors’ knowledge of the study localities. These variables were expected to influence farmers’ knowledge level either positively or negatively and presented in Table 1 below.

**Table 1:** Definition and unit of measurement of variables included in the logit regression model.

variable	code	types	Unit of measurement	Expected sign
**Dependent variable**				
knowledge/awareness level on IK and practices	KnowLevel	Dummy	1 (high knowledge level) if the respondent scored ≥3; 0 otherwise	
**Explanatory variable**				
Age of the household head	AgeHH	Continuous	Measured in years	±
Gender of the household head	GenderHH	Dummy	1 = Male; 0 = Female	±
Education status of the household head	EducHH	Continuous	Measured in years of schooling	±
Farm size	FrmSize	Continuous	Measured in hectare	±
Farming experience	FrmExprs	Continuous	Measured in years of farming	±
Agro-ecology	AgrEcol	Continuous	1 = Highland; 2 = Midland and 3 = Lowland	±

“±” sign represents positive/or negative influences on farmers’ knowledge/awareness on indigenous knowledge and practices

## 3. Results and discussion

### 3.1 Sociodemographic characteristics of the respondents

In total, 288 farmers responded to this study making a response rate of 97.9%. More males (71.5%) were represented than females (28.5%). The age of respondents ranged from 21 to 74 years, where the majority (40.3%) of age groups were in between 35 to 60 years. More than half of the respondents had attended at least primary education. The farm size ranges from 0.18 to 4.56 hectares, resulting in an average landholding size of 1.8 ha, where nearly more than a third of the respondents (42%) have a farm size between 0.5–3.0 ha (Table 2).

**Table 2:** Sociodemographic characteristics of respondents.

variables	Value	Frequency	percent (%)
Sample size	288 out of 294		97.9
Sex of the household head	Male	206	71.5
	Female	82	28.5
Age of the household head (years)	21–34	72	25
	35–60	116	40.28
	60+	100	34.72
Education status of the household head (schooling years)	Illiterate	62	21.53
	Primary education	180	62.5
	Secondary education	46	15.97
Farm size (ha)	≤1.0	78	27.1
	1.1–2.0	99	34.4
	≥2.1	111	38.5
Farming experience (years)	≤5	71	24.7
	6–14	73	25.3
	≥15	144	50

### 3.2 Views of respondents on the existence, current management, and utilization trends of local knowledge systems

The majority (62.9%) of respondents are aware of on the presence of local knowledge systems in their locality. However, the use of local knowledge systems is decreasing in recent periods. Likewise, more than half (55.9%) of the respondents rated poor for the current management trends of local knowledge systems. Accordingly, disrespect to the local knowledge, poor management, and lack of information was the topmost cited factors causing the erosion of local knowledge systems (Table 3). In agreement with the current finding, studies state that land resource degradation, expansion of modern education, disrespect to the local knowledge, among others are assumed to cause for the erosion of local knowledge systems. Moreover, expansion of market-oriented cultivation like monocropping, green revolution, variability in lifestyles where younger fail to learn from fathers, and limited social networking, etc. are key drivers for the erosion of indigenous knowledge and practices [25].

**Table 3:** Views of respondents on presence, utilization, and management trends of traditional ecological knowledge and practices.

Attributes	Response	frequency	Percent (%)
Existence of local knowledge	Yes	181	62.9
	No	107	37.1
Awareness/knowledge/ level	≥3(high)	173	60.1
	<3(low)	115	39.9
Roles of local knowledge systems	Yes	174	60.4
	No	114	39.6
Utilization trends of LK by community	Increasing	116	40.3
	decreasing	172	59.7
Current management status	Excellent	39	13.5
	Fair	88	30.6
	Poor	161	55.9
Threats of local knowledge systems	Disrespect to local knowledge	288	100
	lack of information	265	92
	poor management	283	98.3
	limited roles of LK	21	7.3
	Others, like environmental degradation	18	6.3

### 3.3 The adaptive roles of local knowledge systems to manage environmental disturbances

#### Land resource degradation management practices

The result showed that the local community relies on a wide range of local knowledge systems for natural resources management and biodiversity conservation, medication, manage food insecurity management, climate change adaptation, etc. (Table 4). The result also indicated that soil erosion is a serious hazard, impairing ecosystem health, and agricultural production. As a result, land resource management practices including multiple species management, agroforestry, enclosure, protection of specific habitats, etc. are widely implemented to control accelerated soil erosion, improve soil fertility, and livelihood diversification. Eyewitness during transect walk also revealed that the rugged topography is contoured by terraces and exhibited a mosaic outlined rows constructed to control soil erosion and retain rainwater. These local natural resource management practices have multifaceted contributions in improving biodiversity [28], livelihood diversification [29], and thereby resilience of social-ecological system [3,8].

**Table 4:** Local knowledge systems identified in the Dinki watershed social-ecological system.

Domain	Component	Elements and descriptions
Eco-cognitive domains	conceptual knowledge on the environment	Identification of local climate and landscape conditions: articulating the weather condition and landscape types
		Identification of plant and animal species: naming at least five plant and animal species
		Naming of soil types: naming at least three soil types
		Identification of land-use types: naming of at least three land-use types
		Articulating ecosystem dynamics: able to describe the natural resource base, human population, and climate patterns
Practical domains	natural resources and biodiversity management	Multiple species management (polyculture) practices
		Multiple species management (Agroforestry) practices
		Watershed management (forest management) practices
		Watershed management (enclosure practices):
		Watershed management (soil and water conservation) practices: like stone terraces, water-ways, etc. protection of hillsides from livestock and human entrances protection of specific habitats and total protection of many individual species protection of all individuals of certain species: protection trends of indigenous plant species
		Managing landscape patchiness (rotational grazing) protection of vulnerable life stages: like ban hunting of female ‘*Dikula’*
	adaptive practices	**Land resources management**: on-farm soil and water conservation measures like stone terraces, etc.
		Landscape management through forest and enclosure practices
		Agroforestry practices through live fencing, home garden, and tree garden, etc.
		**Food insecurity management**: improving soil fertility; crop diversity, and redundancy to spread risk; Traditional farming, such as crop-livestock integration, agroforestry, crop rotation, polyculture, water harvesting, etc.; Income diversification through small-scale irrigation, ecosystem-generated goods; storage, etc. Climate change adaptation through weather prediction and early warning system
		**Medication**: using local plant and animal products to treat human and animal diseases.
		**Sustaining social capital:** conflict resolution through elderly institutions, strengthening traditional associations, such as ‘Debo’, ‘Idir’, and other household organizations, pooling through sharing of resources. and technology, etc.
		**Storage:** saving of food items and fodder for future use; strengthening social mechanisms
**S**ociocultural domains	Family kinship knowledge, Cultural, and spiritual skills	**Family knowledge**: Family kinship knowledge for marriage, and resources ownership
		**Conflict resolution**: elderly and religious intuitions
		**Taboos:** govern dos and do not dos
		**Rituals and customs**: customs enacted to guide working days, marriage, daily practices.
		**Rules and regulations**: social rules enacted to teach violators.
		**Cultural values**: beliefs, humility, reciprocity, etc.

NB: The eco-cognitive domain was captured based on the basic conceptual knowledge of the environment and climate; the main activities in the agriculture and natural resource management as well as adaptive practices like medication, storage, etc., were used to explain the practical domains. whereas the family knowledge, cultural, and spiritual skills were used to describe the sociocultural domains of the local knowledge system.

In Dinki watershed, patches of soil and water conservation measures mainly through stone terraces and enclosures are abundant. Such natural resource management practices, particularly focusing on protection of specific habitat like stream banks, riparian corridors, hillsides, etc. are known to reduce surface runoff, maintain trap nutrients, and improve the in-stream condition. As a result, the use of local knowledge for the protection and restoration practices could improve the health of stream habitats and in-stream ecosystems [30,31].

It was found that enclosure practices where humans and animals are restricted to allow natural regeneration are plausible strategies to rehabilitate degraded sites. The practices are also supportive to enhance biological diversity as well as may contribute to livelihood diversification through economic returns. In agreement with the current finding, studies state that enclosures are flourishing strategies practiced to improve biological diversity and ecosystem productivity and practiced in Ethiopian highlands as plausible strategies to rehabilitate degraded sites [32,33].

#### Food insecurity management

Food insecurity could be managed by improving crop yield as well as by reducing household’s vulnerability to climatic shocks. Improving soil fertility and growing of multiple species are most common practices applied to improve farmers’ crop yield. In this aspect, traditional agriculture that involves crop diversity, fallowing, and polyculture is ideal to improve soil fertility and its production system [34]. Similarly, diversification of food sources (agriculture, livestock, wild resources, etc.); water sources (rain, stream, underground) and buildings are a basis to sustain livelihood strategies; thereby resulting in resilient socio-ecological systems [3].

In terms of climate change and variability, rainfall variability and associated shifting of the agricultural calendar, erratic rainfall, recurrent drought, agricultural fatigue, etc. were major signals of climate change and variability identified by the study participants. Local-level weather prediction is considered as an early warning system to manage environmental disturbances. Weather forecasting is supportive of prepared to and reduces the effects of crises. This prediction is highly supportive of adaptation (example: decisions in cropping seasons) and mitigation (reduce the adverse impacts of climate extremes) interventions [35].

#### Sustaining social capital

The role of social capital in strengthening social ties as well as in sustaining peace and security is laudable. For instance, traditional associations such as *‘idir’* and *‘debo’* are gatherings established for group works in natural resource and agricultural activities. These practices are critical to share resources, manpower and technology, and exchange information. Such pooling trends contribute to strengthening social bonds and the basis to manage socioeconomic and environmental crises. In line with this finding, the sharing of resources, labor, and infrastructure was reported to enhance pooling during crises in Southwest Spain [36].

The elderly institutions play a great role in sustainable conflict resolution. It has been successful to strengthen trust and social interactions. A wide range of literature states that indigenous customary laws were the best alternative to manage conflicts when modern dispute systems fail to bring peace and social ties in most Africans [37]. Unlike the modern legal court system, local institutions are instrumental not only in conflict resolution but in reducing revenge killings as well as in restoring cracked social capital. In this aspect, elders’ institutions are known to sustain social capital within families, clans, and communities across Africans. They remained resilient and continue to exist outside the spheres of state influence [38].

#### Traditional medication, storage, and transmission

The findings show that the traditional medication was the mainstay for the local community, and saved their lives before modern health services come to exist. Studies state that more than 80% of the global population relies on traditional knowledge for the medication [39]. Likewise, the Dinki watershed communities are known with their rich sources and application of medicinal plants [40].

Storage of food items and fodder for future sustainable use is widely practiced mitigation tool to lessen the effect of resource limitation. Furthermore, local knowledge systems are transmitted to the next generation mainly through storytelling, proverbs, and songs. Such storage practices of fodder for future use during crises are reported to contribute to enhancing the resilience of herds to food shortages. The same trend of stocking of grains, and cultural values through proverbs, storytelling, and songs was reported in Spain as well [3].

### 3.4 Social mechanisms behind the local knowledge systems

The presence of practical domains is not insurance by itself without social mechanisms that sustain the performances of local knowledge systems [5,10]. The result showed that the family knowledge, elderly institutions, taboos, and totems, rules and regulations, rituals and customs, worldviews, and cultural values were some common social institutions that contribute to support and guide the application of local knowledge systems (Table 4).

#### Family knowledge

It is know-how on family history and origin. Previously this knowledge was critical for land access through the “rist” land tenure system. As people sharing a common ancestor will access free land access, unlike the “gult” land tenure system. More importantly, however, family knowledge has a critical role during marriage and resource conservation. During the Haile Selassie regime, people sharing a common clan will access free land access. The family kinship knowledge is a basis for land resource ownership as well as to differentiate family clans that do not share the same kinship during the marriage [41].

#### Taboos and totems

The finding showed that a wide range of taboos and totems where the study community experience to guide dos and do not dos. For instance, cutting of trees around the church compound is strictly forbidden. Likewise, killing off the female bushbuck is prohibited. In some like Gedamoch sites, large and indigenous tree species are prohibited to cut. Similarly, studies state that taboos are social mechanisms that restrict the use of some or whole parts of a species or habitat and effective to nature conservation [5,11].

#### Rules and regulations

The result showed that natural resources management and agricultural activities are principally guided by local rules and regulations. For instance, during intensive soil erosion, the existing soil and water conservation practices will be strengthened, where rules will be established or improved for their effectiveness. The elderly institutions or watershed management council will enforce the rules. Violation to obey rules leads to punishment in kind, cash as well as exclusion from the public services in extreme cases. In line with this finding, previous studies state that informal institutions are valid for local people and responsible to set rules and regulations for zonation and sustainable utilization of natural resources [42].

#### Rituals and customs

It was found that the majority of ceremonies have a religious background, where food items, marriage, holidays, markets, and working days are defined based on a religious perspective. The traditional association is established to help the community in labor as well as during crises and marriage. Accordingly, “*Idir*” and “*Debo*” are two of the most customary traditional associations contributing to the social welfare of the rural. The former is established to help member parties during crises, like illness, death, crop failure, and so on. The latter is the most conspicuous in this locality where peoples of working-age form teams to work resource management and agricultural works. These group works are usually accompanied by traditional food and drinks as well as proverbs and songs are thought to facilitate group works. The same trend was also reported in Spain [3].

#### Worldviews and cultural values (beliefs, share, respect, reciprocity, humility, etc.)

Worldviews and cultural values through reciprocity, sharing, and respect are the basis to acquire and transmit indigenous practices as well as to nature conservation. In agreement with the current finding, studies state that the worldviews and beliefs influence the perception of the surrounding environment, and the basis to interpret observations from nature [5,8,11] In Ethiopia, including in the study area, churches are marked with patches of forests often protected as sacred grooves remained preserved for centuries [43]. It is highly historic and spiritual to plant and preserve trees around churches and monasteries (Genesis 2: 16–17; Genesis 6). In effect, growing and protection of plants are rooted from a biblical perspective; where their presence signifies all the paths that Christians pass from early creation to the end of this world [44]. Thus, Church forests provide spiritual and ecological services (example: shade, freshwater) to their surrounding communities and their contribution to mountain management is paramount [43,44].

### 3.5 Determinant factors that influence the acquisition of local knowledge systems

The logistic regression analysis was performed to assess the impact of a set of predictors on the likelihood of respondents who would acquire the eco-cognitive, practical and normative domains of the local knowledge systems. The result of the logistic regression analysis showed that the explanatory variables, such as age, gender and education were statistically significant in cognitive, practical and normative domains, the farming experience was significant in practical and normative domains as well as the farm size was significant in practical domain of indigenous knowledge and practices. It suggests that the model was able to distinguish between the respondents who reported and did not report on their knowledge (indigenous) level (Table 5).

**Table 5:** Summary of logistic regression predicting the local knowledge acquisition in the Dinki watershed socio-ecological system, Ethiopia.

Explanatory variables	Domains of local knowledge systems								
	Eco-cognitive			Practical			Sociocultural/normative		
	Coefficient	Std. error	Odds ratio	Coefficient	Std. error	Odds ratio	Coefficient	Std. error	Odds ratio
AgroEcol	0.19	0.26	1.21	0.28	0.18	1.33	0.28	0.24	1.33
Gender (1) HH	2.51***	0.49	12.3	2.19***	0.66	9.53	2.26***	0.47	9.53
AgeHH	1.67***	0.51	5.30	1.81***	0.42	4.39	1.48***	0.45	4.39
FrmExprs	0.6	0.50	1.82	1.86**	0.67	3.11	1.13*	0.44	3.11
FrmSize	0.72	0.49	2.05	1.99**	0.50	0.49	0.17	0.45	1.19
EducHH	2.8***	0.59	16.84	-4.68***	0.91	1.58	1.37**	0.47	3.93

*p-value significant at 10%

**p-value significant at 5%

***p-value significant at 1%.

**Fig 2:**
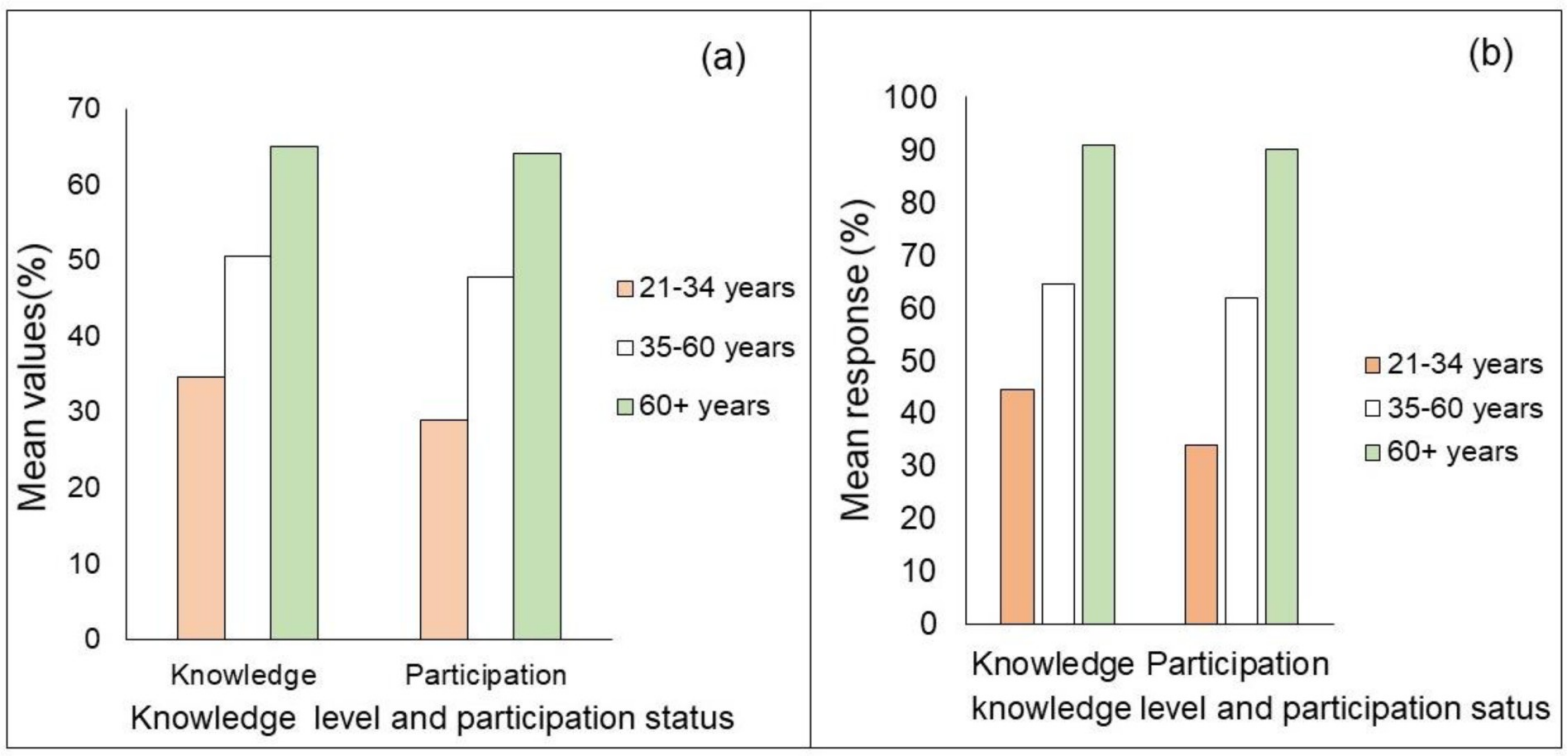
Intergenerational variation in knowledge and participation status in practical (a) and sociocultural (b) domains of local knowledge systems.

The logistic regression analysis indicated that the age of the household head has a positive and significant effect on farmers’ knowledge acquisition and transmission at a 1% significance level in all aspects of LK. Accordingly, for each one-year increase in age, the odds ratio will raise 5, 4, and 4 times to acquire eco-cognitive, practical, and socio-cultural domains of LK, respectively (Table 5). The result of one-way ANOVA also revealed statistically significant difference among age groups in eco-cognitive domain. Likewise, the results of the Tukey Post-hoc test confirmed that the mean difference of elders (age>60 years) was statistically significant in all eco-cognitive aspects of LK. Moreover, the result of the Chi-square statistics revealed that the majority of practical and normative domains showed statistically significant differences, where both knowledge and participation status in practical (Fig 2A) and normative (Fig 2B) domains were increased with the age of respondents.

The age of the household head was positively correlated with the knowledge level, where the age of the respondents increases their corresponding knowledge acquisition increases. This could suggest that older farmers might have accumulated a body of knowledge on their environments, changing conditions, and might have acquired a multifaceted knowledge system that could support them to adapt to and survive. In agreement with the current finding, a study in Southern Ethiopia found that the young participants were limited in their knowledge of agroforestry practices than the middle and old-aged participants [26].

**Fig 3:**
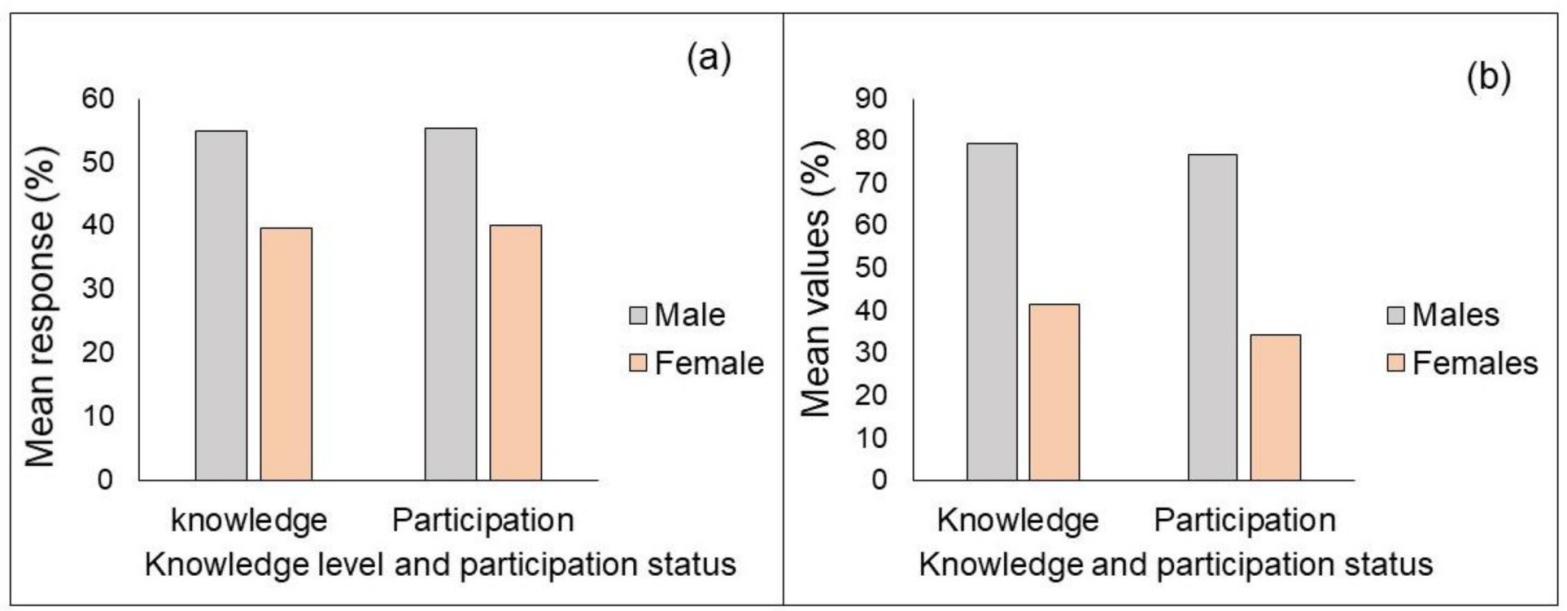
Gender-based variation in knowledge and participation status in practical (a) and sociocultural (b) domains of local knowledge systems.

As expected, the gender of the respondents was found to have a positive and significant correlation with farmers’ knowledge acquisition at 1% significant level. Accordingly, for each one male added in the community, the odds ratio will raise 12, 10, and 10 times to acquire eco-cognitive, practical, and sociocultural domains, respectively (Table 5). The t-test result revealed a statistically significant means score difference between males and females in all eco-cognitive knowledge components (P<0.001). The t-test result showed that females were inferior to males in identifying components of eco-cognitive knowledge. Similarly, the chi-square statistics result revealed a statistically significant difference between males and females in their knowledge and participation in practical domains (Fig 3A) and most normative (Fig 3B) domains. This suggests that these cultural and religious ceremonies are celebrated equally in both males and females in the Dinki watershed socio-ecological system.

It might be the effect of cultural influence where the females in most rural Ethiopia are restricted to in-door tasks, the knowledge and participation of females were inferior to males. In rural Ethiopia, the majority of outdoor activities, including natural resources management practices are predominantly performed by males. This finding agrees with the previous study in Southern Ethiopia that states females’ knowledge and participation in agroforestry practices were minimal than males [26]. Likewise, a study in Northern Ghana showed that females’ awareness of the roles of traditional ecological knowledge in ecosystem management were limited than males [11].

The education level of the respondents showed variable correlations with the acquisition of local knowledge systems. It was found to have a positive and significant correlation with eco-cognitive but negative and significant correlation with practical domains, each at a 1% significance level. However, it exhibited a positive and significant correlation with sociocultural domains at a 5% significance level. The logit analysis revealed that for each one-year increase in schooling, the odds ratio will raise 17 and 4 times to acquire eco-cognitive and sociocultural domains, respectively. However, it will drop 2 times to acquire practical domains of LK (Table 5).

The chi-square statistics were also revealed a statistically significant difference between education and all domains of LK. However, education, particularly primary education was found to have favored acquisition of eco-cognitive and sociocultural domains. In the practical domain, primary education was promising to favor knowledge acquisition. However, secondary education level was found even inferior to the illiterate level in acquiring the practical domain of LK (Table 6).

**Table 6:** Effects of schooling on eco-cognitive, as well as on knowledge and participation of practical and sociocultural of local knowledge systems.

Education status of the household head	Mean values (%) in each domain of local knowledge systems				
	Eco-cognitive domain	Practical domain		Sociocultural domain	
		knowledge	Participation	Knowledge	participation
Illiterate	19	49	48	43	38
Primary education	63	60	58	83	82
Secondary education and above	33	22	27	46	33

A wide range of literature reports controversial results on the association of schooling with indigenous knowledge acquisition and transmission. Schooling is argued to significantly reduce the time children spent along with their parents resulting in the intergenerational gap [45]. A wide range of literature argues that literacy and increasing years of schooling significantly affect the acquisition and transmission of local knowledge systems [46,45].

On the other hand, the United Nations Education for Science and Nature organization (UNESCO) argues that education can result in both erosions as well as the development of indigenous knowledge and practices [47]. In this study, households who attended primary education were superior in acquiring local knowledge system (Table 6). In line with the current finding, although increasing years of schooling significantly erode local knowledge [46,45], a minimum of primary education was identified promising to record and conserve medicinal knowledge in western Nigeria [48].

When formal education is a limiting factor to the acquisition and transmission of local knowledge, time and curriculum are the key factors attributed to causing intergenerational gaps. The majority of local knowledge is acquired through hands-on practices that involve more time to spend along with custodians. In this aspect, schooling limits the time children spent in the community, and the school curriculum is the second principal factor to limit indigenous knowledge acquisition. It is often designed based on western science that undervalues local culture and knowledge [25,46].

On the other hand, formal education can create a promising setting for learning to promote local knowledge by empowering custodians’ interest and memorization skills, like by recording medicinal plants [49]. In this regard, contextualized learning favors the acquisition of integrated curriculum [45] through integrating education with local cultures. It is effective to design locally pertinent science curriculum, thereby contributing to societal developments through economic development and environmental responsibility. Such integration is critical to address factors that limit local knowledge acquisition and transmission [46].

## 4. Conclusion

Despite the current environmental degradation, the result showed the existence of remnants of local knowledge, indicating their resilience to environmental disturbances. The intergenerational gap was prevalent, where most practices are held by elders. Likewise, the majority of natural resource management practices were male-dominated, indicating the cultural influence that restricts females to domestic tasks. Furthermore, it might be the influence of the school curriculum, farmers who attended beyond primary education were inferior in most local knowledge systems. In general, however, local knowledge systems are a plausible option and their use needs to be enhanced to promote farmers’ adaptive potential to a multitude of disturbances. Moreover, place-based education is required to integrate the local knowledge systems into the school curriculum. Likewise, local-level decisions should participate in custodians to maintain sustainable and resilient socio-ecological systems.

## Supporting information

S1 Appendix (DOCX)
