# Supplementary material for: The role of local knowledge in enhancing the resilience of dinki watershed social-ecological system, central highlands of Ethiopia
Source: PLoS One. 2020 Sep 4;15(9):e0238460. doi: 10.1371/journal.pone.0238460 (PMC7473574; doi:10.1371/journal.pone.0238460)
Supplement: S1 Appendix — (DOCX) [file pone.0238460.s001.docx]

## Appendix I. Items related to the eco-cognitive, practical and socio-cultural domains of local knowledge systems

|  | Variables raised to assess the eco-cognitive, practical and socio-cultural domains of local knowledge systems | 1=yes;0=n0 |
| --- | --- | --- |
| 1 | I can describe the topography and climate condition of my locality |  |
| 2 | I can mention more than five animal and plant species |  |
| 3 | I can mention more than three soil types in my locality |  |
| 4 | I can mention more than three land-use types in my locality |  |
| 5 | I can explain the dynamics of the ecosystem during the last decades |  |
| 6 | I often experience to protect specific habitats like river banks, hillsides, etc. |  |
| 7 | I often used to protect indigenous plants like Zigba, Koso, Tid, etc. |  |
| 8 | I know some vulnerable life stages like female dikula needs to be protected |  |
| 9 | I often used to experience multiple cropping practices |  |
| 10 | I often experience agroforestry practices |  |
| 11 | I used to apply rotational grazing |  |
| 12 | I often experience forest management practices |  |
| 13 | I understand the roles of an enclosure |  |
| 14 | I used to experience SWC practices on my farmland |  |
| 15 | I know some land resources degradation malmanagement practices |  |
| 16 | I know some common food insecurity management practices |  |
| 17 | I know tradition human and animal medication practices |  |
| 18 | I understand that social capital needs to be strengthened |  |
| 19 | I know some storage practices of resources for future use |  |
| 20 | I know some basic family knowledge at least for my clans |  |
| 21 | I have some resolution skills and involved in an elderly institution |  |
| 22 | I know some taboos in my locality |  |
| 23 | I know some ritual and customs like market and working days,  family kinship knowledge, traditional associations in my locality |  |
| 24 | I know some social rules enacted to guide daily activities |  |
| 25 | I know the cultural values of my locality like beliefs, respect, humility, etc. |  |
|  | Average |  |
